# Heat wave effects on the behavior and life-history traits of sedentary antlions

**DOI:** 10.1093/beheco/araa085

**Published:** 2020-09-22

**Authors:** Krzysztof Miler, Daniel Stec, Marcin Czarnoleski

**Affiliations:** 1 Institute of Systematics and Evolution of Animals, Polish Academy of Sciences, Sławkowska, Kraków, Poland; 2 Institute of Zoology and Biomedical Research, Faculty of Biology, Jagiellonian University, Gronostajowa, Kraków, Poland; 3 Institute of Environmental Sciences, Faculty of Biology, Jagiellonian University, Gronostajowa, Kraków, Poland

**Keywords:** antlion, *Euroleon nostras*, heat wave, *Myrmeleon bore*, thermal stress, trap-building

## Abstract

Research on the behavioral responses of animals to extreme weather events, such as heat wave, is lacking even though their frequency and intensity in nature are increasing. Here, we investigated the behavioral response to a simulated heat wave in two species of antlions (Neuroptera: Myrmeleontidae). These insects spend the majority of their lives as larvae and live in sandy areas suitable for a trap-building hunting strategy. We used larvae of *Myrmeleon bore* and *Euroleon nostras*, which are characterized by different microhabitat preferences—sunlit in the case of *M. bore* and shaded in the case of *E. nostras*. Larvae were exposed to fluctuating temperatures (40 °C for 10 h daily and 25 °C for the remaining time) or a constant temperature (25 °C) for an entire week. We found increased mortality of larvae under heat. We detected a reduction in the hunting activity of larvae under heat, which corresponded to changes in the body mass of individuals. Furthermore, we found long-term consequences of the simulated heat wave, as it prolonged the time larvae needed to molt. These effects were pronounced in the case of *E. nostras* but did not occur or were less pronounced in the case of *M. bore*, suggesting that microhabitat-specific selective pressures dictate how well antlions handle heat waves. We, thus, present results demonstrating the connection between behavior and the subsequent changes to fitness-relevant traits in the context of a simulated heat wave. These results illustrate how even closely related species may react differently to the same event.

## Teaser

Animals must cope with what the environment throws at them. When that is a heat wave, sedentary insect predators, such as antlions, either reduce hunting activity and suffer fitness-relevant consequences or remain actively hunting and successfully buffer against harsh conditions. The type of displayed reaction depends on the ecological and evolutionary characteristics of a species experiencing a heat wave.

## INTRODUCTION

An increase in the frequency and intensity of heat waves has been observed around the globe, and there is a growing consensus that this is one of the key features of climatic changes in the Anthropocene (Coumou and Rahmstorf 2012; Ummenhofer and Meehl 2017; Perkins-Kirkpatrick and Gibson 2017; Chapman et al. 2019). Biological research focusing on the importance of heat waves indicates their far-reaching impact on the biotic environment. For instance, both subcontinental and marine heat waves have been shown to markedly alter the ecosystem structure, composition, and function (Ruthrof et al. 2018; Sanford et al. 2019). On an organismal level, heat waves were demonstrated to induce the production of heat shock proteins (Olabarria et al. 2016) and change hematological parameters (Pegado et al. 2020), as well as negatively impact fitness-relevant traits, such as body condition and mortality (Leung et al. 2017; Abarca et al. 2019) or sperm quality and function (Hurley et al. 2018; Sales et al. 2018). However, the susceptibility of animals to all these effects largely depends on their immediate behavioral responses to heat waves, which ultimately dictate the extent to which essential traits, such as longevity or fertility are affected (Huey et al. 2012; Beever et al. 2017). This is especially true because of slow physiological acclimation to increased temperature demonstrated by many animals (Bar-Ziv and Scharf 2018; Cooper et al. 2020). The issue of behavioral responses to heat waves, surprisingly, is much less often studied, typically being only anecdotal and supplementary to other types of research (Buchholz et al. 2019).

Heat wave-related behavioral modifications can be considered adaptive or maladaptive depending on their fitness outcomes. For instance, in response to heat, ladybeetles increase their egg-laying frequencies, which is probably beneficial (Sentis et al. 2015), whereas grasshoppers start ignoring predation cues, which is detrimental (Schmitz et al. 2016). Behavioral thermoregulation, one of the most widespread and researched responses to heat (Funghi et al. 2019), provides an example of an adjustment that may just tip the scales from loss to gain in the context of animal fitness. For example, some birds are predicted to fail at balancing out the avoidance of heat and the severity of dehydration, leading to catastrophic mortality (McKechnie and Wolf 2010). On the other hand, social insects, such as ants, can find such a balance and cope very well with thermal extremes (Andrew et al. 2013). Recently, this fragile relationship was demonstrated in possums, which show effective behavioral thermoregulation but only up to a certain point (Turner 2020). Thus, heat can be difficult to cope with by behavioral means even within a relatively small range of temperatures, especially in species characterized by conserved, locally adapted thermal niches (Bennett et al. 2019). For those species, behavioral means have no chance of providing more time for later genetic adaptation and/or range modifications—which are crucial in changing environments (Tuomainen and Candolin 2011; Gunderson and Stillman 2015). Therefore, current research must uncover the ways in which animals react behaviorally to extreme weather events, such as heat waves (Buchholz et al. 2019).

Here, we used predatory trap-building antlions (Neuroptera: Myrmeleontidae) to investigate their behavioral response to a simulated heat wave and its subsequent effects. Antlions spend the majority of their lives as sit-and-wait predatory larvae characterized by low dispersion and a sedentary lifestyle, during which most species inhabit sandy areas where they construct pitfall traps to hunt small invertebrates, such as ants (Scharf and Ovadia 2006; Turza et al. 2020). Thus, antlions seem to be prone to strong selection by various abiotic features, including temperature, which makes them ideal models for the present study. Previous research has demonstrated temperature-related changes in antlion activity, as well as their generally high thermal resilience. For example, they were found to bury deeper in the sand to avoid heat and, thus, fail at prey capture attempts above 35 °C (Green 1955), as well as to entirely cease pitfall trap construction above 42 °C (Youthed and Moran 1969). Interestingly, antlion species differ in their microhabitat preferences, presumably reflecting on their local thermal niche adaptations. Here, we used two species of antlions, *Myrmeleon bore* characteristic of sunlit areas and *Euroleon nostras* occurring in shaded areas, to account for potential interspecific differences in heat wave tolerance between the species. Additionally, because larval instars of antlions differ in their behavior (Alcalay et al. 2014), we used the second and third instars of both species to account for potential developmental differences between instars. We exposed antlions to either fluctuating temperature (40 °C for 10 h daily and 25 °C for the remaining time) or constant temperature (25 °C) for a week. These thermal conditions were chosen to simulate an extreme weather event in the form of a heat wave and more typical summertime weather.

Our study species were previously demonstrated to experience differing levels of temperature fluctuations in nature, that is, relatively higher in *M. bore* and lower in *E. nostras* (Lackinger 1973; Abraham 2003). Additionally, both of these species show an increase in some activity parameters at 35 °C when compared with lower temperatures (Klokočovnik et al. 2016; Antoł et al. 2018). Therefore, we considered *E. nostras* larvae found in shaded microhabitats to be much more susceptible to our hypothesized effects. We expected that heat wave exposure (i.e., fluctuating temperature treatment) would increase the immediate mortality of antlions (hypothesis 1) and significantly impair their hunting activity, leading to weight loss (hypothesis 2). We also expected that, after experiencing a heat wave, second instars would need more time to molt, whereas third instars would be forced to overwinter in worse body state (hypothesis 3).

## METHODS

In July 2019, we collected second and third instar larvae of *M. bore* and *E. nostras* antlions in the Błędowska Desert (Poland, coordinates: 50º20’24′’N, 19º32′20′’E). The species are easily distinguished at sight based on body coloration and lack of microhabitat overlap (Badano and Pantaleoni 2014). The instars in both species can be differentiated based on body dimensions, mainly head size and shape (Devetak et al. 2005; Jingu and Hayashi 2018). After transportation to the laboratory, larvae were weighed to the nearest 0.001 g on an electronic balance and then individually housed in labeled plastic cups (7 cm in diameter and 15 cm in height) half filled with sand. Larvae were then left for the night to acclimate. The next morning, larvae were sorted into four groups based on species identity (*M. bore* vs. *E. nostras*) and instar stage (second vs. third). Individuals from each group were allocated to two experimental treatments (30 larvae per group per experimental treatment) with either fluctuating or constant temperature. The two experimental treatments were generated in thermal cabinets, set either to 40 °C between 9 AM and 7 PM and then to 25 °C for the remaining time (fluctuating treatment) or to a constant 25 °C (constant treatment). The light and dark cycle was set such that daytime occurred between 9 AM and 7 PM and nighttime occurred between 7 PM and 9 AM in both cabinets. Treatments lasted for 7 full days (hereafter, “simulation”) and involved 240 larvae (2 species × 2 instar stages × 2 experimental treatments × 30 individuals).

During the simulation, the activity status of larvae was checked each day around noon. We noted whether a given larva was “active” or “inactive”. Active larvae were considered to 1) maintain pitfall traps (i.e., functional and undisturbed) and 2) be visible (i.e., mandibles protruding from the bottom of the trap). The activity of larvae determined whether a given individual was fed or not in line with previous reports of hindered feeding in inactive larvae (Green 1955; Youthed and Moran 1969; Cain 1987). Thus, each active larva was given a single prey item, a live *Lasius niger* ant worker, and each inactive larva was checked for signs of life (i.e., dug out and gently prodded using forceps). Live inactive larvae were left in their cups, whereas dead inactive larvae were discarded and marked as dead. After the simulation, larvae were weighed to the nearest 0.001 g on an electronic balance and then placed on shelves in the laboratory (under constant 23 °C temperature, 50–70% relative humidity, and natural light:dark cycle). For the next 70 days, until the end of September, each day around noon, the cups were checked for the activity status of larvae. All active larvae were given a single prey item, as during the simulation, and all inactive larvae were checked for signs of molting in the case of second instars and pupating in the case of third instars (i.e., dug out for verification). Inactive larvae not undergoing molting or pupating were left in their cups, whereas molting or pupating inactive larvae were discarded and marked accordingly. All larvae remaining in the experiment on the last day were weighed to the nearest 0.001 g on an electronic balance and marked as entering winter. The experiment ended at the time of year when, in the field, weather conditions already force antlions to overwinter, that is, cease foraging activity.

Statistical analyses were conducted using the statistical programming language R (R Core Team 2020) with the mdscore, emmeans, and ggplot2 packages. To test hypothesis 1 (heat wave exposure will increase the immediate mortality of antlions), we compared the mortality of antlions using a generalized linear model with binomial distribution (1—died and 0—lived), logit link function and three fixed factors, the species (*M. bore* vs. *E. nostras*), instar stage (second vs. third), and experimental treatment (constant vs. fluctuating). The model included interactions of these factors. To address hypothesis 2 (heat wave exposure will significantly impair antlion hunting activity, leading to weight loss), we used general linear models. First, we compared the body mass before the experiment with the species (*M. bore* vs. *E. nostras*) and instar stage (second vs. third) as fixed factors. The model included species × instar stage interaction. Then, we analyzed the number of days of activity during the simulation and the change in body mass over the simulation, both with species (*M. bore* vs. *E. nostras*), instar stage (second vs. third), and experimental treatment (constant vs. fluctuating) as fixed factors. The model included interactions of these factors. To test hypothesis 3 (after heat wave exposure, second instars will need more time to molt, whereas third instars will be forced to overwinter in worse body state), we again used general linear models. We analyzed the number of days to molt after the simulation (in second instar larvae) and the final body mass after the simulation (in third instar larvae), both with species (*M. bore* vs. *E. nostras*) and experimental treatment (constant vs. fluctuating) as fixed factors. These models included species × experimental treatment interactions. In each general linear model, we used post hoc Tukey test comparisons.

## RESULTS

The analysis of mortality revealed that all interactions were nonsignificant and did not improve the fit of the model; hence, they were removed from the final model. Significant differences were found between species (Wald’s χ ^2^ = 3.92, *P* = 0.048; higher mortality in *E. nostras*), instar stages (Wald’s χ ^2^ = 6.43, *P* = 0.011; higher mortality in second instars), and experimental treatments (Wald’s χ ^2^ = 5.66, *P* = 0.017; higher mortality under fluctuating treatment), with the highest mortality in the second instars of *E. nostras* exposed to heat wave (7 dead out of 30 larvae).

**Figure 1:**
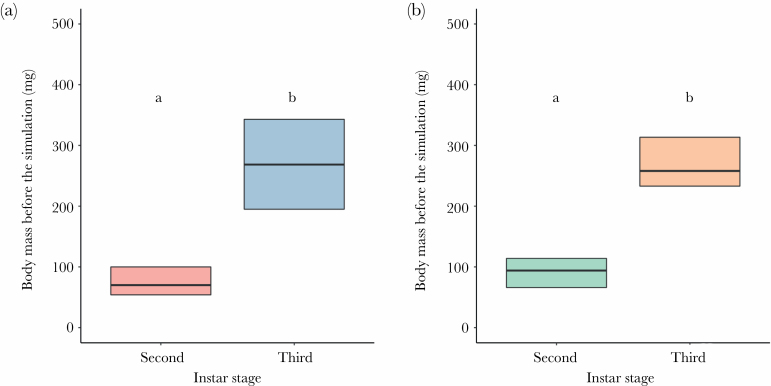
The body mass of larvae before the simulation. Panel A shows the results for *M. bore*, and panel B shows the results for *E. nostras*. Bold lines inside boxes indicate mean values, and boxes indicate quartiles. The number of tested individuals was *N* = 30 for each type of larvae. Small letters above boxes indicate significance as yielded by post hoc Tukey comparisons.

The results for the body mass before the experiment showed that the effect of the species × instar stage interaction was nonsignificant. Body mass depended on the instar stage (*F*_1,224_ = 525.23, *P* < 0.001; third instars heavier than second instars) but not on the species (*F*_1,224_ = 2.99, *P* = 0.085; Figure 1).

**Figure 2:**
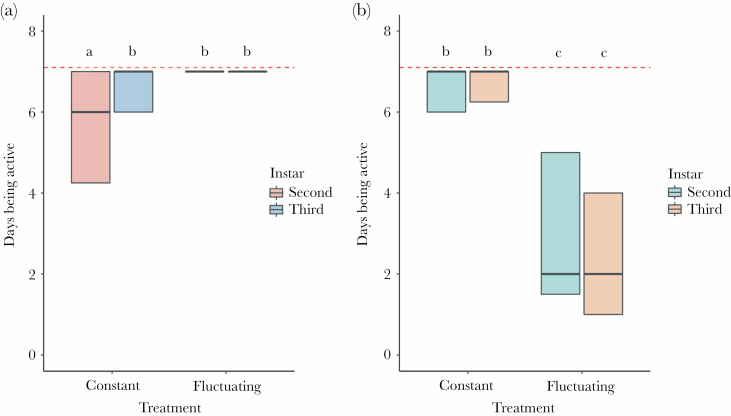
The number of days of activity during the simulation. Panel A shows the results for *M. bore*, and panel B shows the results for *E. nostras*. Bold lines inside boxes indicate mean values, and boxes indicate quartiles. Dashed lines indicate maximum values (full 7 days of the simulation). The number of tested individuals ranged from 23 to 30 for each type of larvae tested (individuals who died during the experiment were excluded). Small letters above boxes indicate significance as yielded by post hoc Tukey comparisons.

**Figure 3:**
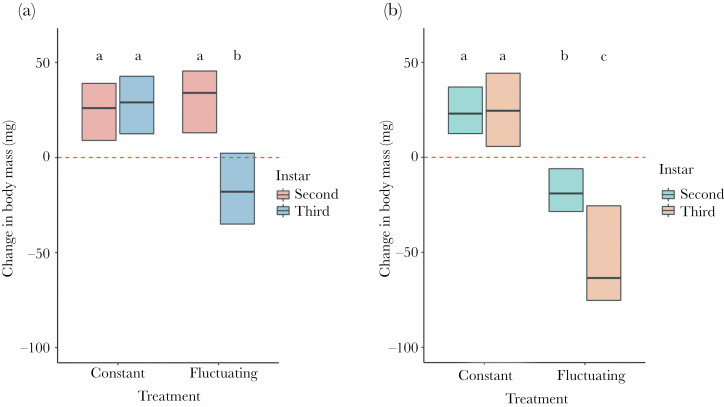
The change in body mass over the simulation. Panel A shows the results for *M. bore*, and panel B shows the results for *E. nostras*. Bold lines inside boxes indicate mean values, and boxes indicate quartiles. Dashed lines indicate the point of no gain and no loss (change in body mass equal to 0). The number of tested individuals ranged from 23 to 30 for each type of larvae tested (individuals who died during the experiment were excluded). Small letters above boxes indicate significance as yielded by post hoc Tukey comparisons.

The results for the number of days of activity during the simulation were complex (Table 1). In *M. bore*, activity levels dropped slightly in the second instars under the constant treatment, whereas, in *E. nostras*, a severe drop was observed under the fluctuating treatment, especially in third instars (Figure 2). Additionally, the analysis of the change in body mass over the simulation showed that it depended on the species, instar stage, and experimental treatment (Table 2). In general, larvae of both species, irrespective of instar stage, gained mass in the constant treatment, but the third instars of *M. bore*, as well as the second and third instars of *E. nostras* lost weight in the fluctuating treatment (Figure 3). Body mass change was most severe in the case of third instar *E. nostras* larvae.

**Table 1:** The results of a general linear model for the number of days of activity during the simulation

Model component	*F*_1,220_	Deviance	Residual deviance	*P*
Null			1044.26	
Species	78.83	162.850	881.41	<0.001
Instar stage	0.45	0.931	880.48	0.503
Treatment	59.18	122.246	758.24	<0.001
Species × treatment	136.74	288.434	469.80	<0.001
Species × instar stage	3.39	6.744	463.06	0.067
Instar stage × treatment	4.16	8.593	454.47	0.043

**Table 2:** The results of a general linear model for the change in body mass over the simulation

Model component	*F*_1,220_	Deviance	Residual deviance	*P*
Null			351565	
Species	31.76	21385	330180	<0.001
Instar stage	55.59	37430	292750	<0.001
Treatment	148.79	100188	192562	<0.001
Species × treatment	38.08	25644	166918	<0.001
Species × instar stage	0.01	9	166909	0.907
Instar stage × treatment	27.87	18769	148140	<0.001

**Figure 4:**
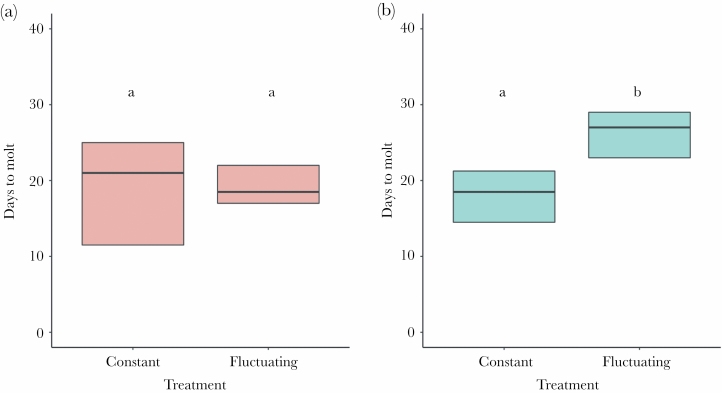
The number of days to molt after the simulation in second instar larvae. Panel A shows the results for *M. bore*, and panel B shows the results for *E. nostras*. Bold lines inside boxes indicate mean values, and boxes indicate quartiles. The number of tested individuals ranged from 23 to 30 for each type of larvae. Small letters above boxes indicate significance as yielded by post hoc Tukey comparisons.

**Figure 5:**
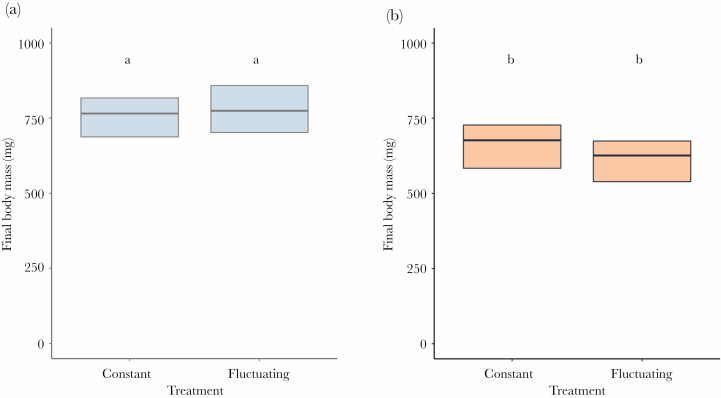
The final body mass of third instar larvae. Panel A shows the results for *M. bore*, and panel B shows the results for *E. nostras*. Bold lines inside boxes indicate mean values, and boxes indicate quartiles. The number of tested individuals ranged from 29 to 30 for each type of larvae. Small letters above boxes indicate significance as yielded by post hoc Tukey comparisons.

During the time period after the simulation and until the experiment was terminated at the end of September (i.e., over 2 months), antlions were kept in the same laboratory conditions and fed whenever active. All second instars molted at some point in this period. The results for the number of days to that event showed that it depended on the experimental treatment (*F*_1,104_ = 11.04, *P* = 0.001) but not on species (*F*_1,104_ = 1.27, *P* = 0.262). A significant effect of the species × experimental treatment interaction (*F*_1,104_ = 8.57, *P* = 0.004) indicated that heat resulted in an increased time to molt only in *E. nostras* larvae (Figure 4). No third instar larva pupated until the end of the experiment, and, thus, all were considered forced to overwinter. The results for the final body mass after the experiment showed that it depended solely on the species (*F*_1,115_ = 28.00, *P* < 0.001), with *E. nostras* being lighter than *M. bore* (Figure 5). The experimental treatment (*F*_1,115_ = 0.23, *P* = 0.629) and the effect of the species × experimental treatment interaction (F_1,115_ = 1.42, p = 0.236) were nonsignificant.

## DISCUSSION

The behavioral response to a heat wave demonstrates the ability of an animal to cope with these extreme thermal conditions and constitutes a bridge between a heat wave as an event and the fitness-relevant consequences of this event (Buchholz et al. 2019). Here, we showed that microhabitat-specific selective pressures might dictate how well antlions handle heat waves. We found partial confirmation that heat wave exposure increased the immediate mortality of antlions (hypothesis 1)—an effect that occurred only in one of the two studied species, *E. nostras*, characteristic of shaded areas in nature. Furthermore, we found partial confirmation that heat wave exposure impaired antlion hunting activity, leading to weight loss (hypothesis 2). Specifically, the shade-preferring larvae of *E. nostras* were often inactive under heat, irrespective of the instar stage (Figure 2). The activity levels of these larvae corresponded to the body mass change over the course of the simulation. Indeed, both instars lost weight over the simulation (Figure 3), as could be expected from the fact that they were not fed. However, the sun-preferring larvae of *M. bore* were highly active during the simulation irrespective of the instar stage (Figure 2). In fact, second instars of this species even showed a slight drop in activity in the constant treatment. Surprisingly, weight loss occurred in the third instars despite their high activity (and feeding) under heat (Figure 3). This effect may stem from the fact that both instar stages were fed with the same amount of prey, which could potentially be sufficient for the second instars but not for the third instars. Indeed, food availability was suggested for antlions to be more constraining than temperature (Arnett and Gotelli 1999). Unfortunately, this suggestion was made based on an experiment in which levels of the food and temperature factors were likely disproportional, which makes conclusions less certain. In any case, here, between-species differences in activity and body mass change cannot be attributed to interspecific differences in initial body mass before the experiment, as the mass of larvae was similar within instars (Figure 1).

Moreover, in terms of second instars needing more time to molt and third instars forced to overwinter in worse body state after heat wave exposure (hypothesis 3), we also found partial confirmation. Heat wave exposure increased the time to molt in second instars of *E. nostras* but not of *M. bore* (Figure 4). This further demonstrates that, for the sun-preferring larvae of *M. bore*, the effects of the simulated heat wave were much less severe. Against our predictions, however, we found that the third instars of both species seemed able to recover after a heat wave. At the end of the season, over 2 months after the simulation, larval body mass did not differ between experimental treatments (Figure 5). Although this was not surprising in the case of *M. bore* larvae, the complete disappearance of earlier severe effects in the case of *E. nostras* was staggering. It is likely that their regaining capacity is possible due to the extremely lengthy larval life of antlions, which may last up to 3 years (Scharf and Ovadia 2006), during which larvae undergoing development may be able to compensate for various environmental influences (Scharf et al. 2011). The mechanism and costs of such compensatory growth in antlions have rarely been studied. There is some evidence to suggest that the mechanism involves alterations in metabolic rates and that the costs involve a trade-off between growth and starvation. Specifically, antlions were demonstrated to be able to increase and decrease their metabolism under favorable and unfavorable environmental conditions (Lucas 1985; Matsura and Murao 1994). Presumably, the postsimulation period in the laboratory was relatively favorable for *E. nostras* third instars, especially after a period of unfavorable conditions (the fluctuating treatment) and enabled them to facilitate their metabolism and growth. However, such compensation may actually decrease later starvation endurance of larvae (Scharf et al. 2009), possibly critically important during the following overwintering. Whether winter survival of antlions with or without prior heat wave experience would differ is an open question.

Note that the constant and fluctuating treatments, which were utilized here, matched the natural conditions experienced by the two antlion species, that is, more constant in *E. nostras* and more fluctuating in *M. bore* (Lackinger 1973; Abraham 2003). This means that the constant treatment with lower temperature (25 °C) was better for *E. nostras* larvae, whereas the fluctuating treatment with higher temperature (40 °C during the day and 25 °C during the night, averaging 31.5 °C), in turn, was better for individuals of *M. bore*. Certainly, one could attempt to differentiate between the effects of heat waves and higher average temperatures associated with heat waves by including another treatment, that is, constant 31.5 °C. We, however, considered that such treatment would be suboptimal for both antlion species, which is why it was not used.

Other taxa seem to show similar reactions to heat waves to those reported for the *E. nostras* antlions herein. For example, in *Sitobion* grain aphids and Western black widow spiderlings, exposure to heat waves prolonged their developmental time (Jeffs and Leather 2013; Johnson et al. 2019). In fact, the disruption of life histories, especially in terms of life stage shifts, is postulated to be one of the main consequences of extreme climatic events (Wingfield et al. 2016). Nevertheless, the behavior of *M. bore* antlions does not entirely fit into this picture. These larvae, similar to other larvae occurring in sunlit environments, are highly heat tolerant (Miler et al. 2019) and, as such, may possess unique coping mechanisms enabling them to lower the adverse effects of extreme heat (such as diel movement in the sand, see, e.g., Cain 1987 and Marsh 1987). These species could be well preadapted for the current global increases in the frequency and intensity of heat waves. Indeed, as demonstrated for *Cueta lineosa* antlions inhabiting sunlit desert areas, they show superb survival rates, foraging success, and starvation endurance in extremely harsh environments (Rotkopf et al. 2012; Barkae et al. 2017).

Interestingly, for the dragonfly–newt system, heat waves have been demonstrated to affect trophic interactions by diminishing predation rates (Smolinský and Gvoždik 2014). Antlions are involved in a trophic interaction or, more specifically, a predator–prey interaction with ants, which are their main prey (Morrison 2004; Barkae et al. 2017; Jingu and Hayashi 2018; Turza et al. 2020). It would be interesting to investigate how the effects of heat waves, demonstrated here, shape this particular system in terms of predation rates. It is especially intriguing because sand-dwelling ants, such as the thermal specialists of the genus *Cataglyphis*, are well known for their extraordinary thermal resistance (Wehner et al. 1992; Pfeffer et al. 2019). As we showed here, the hunting activity of antlions may be hindered under prolonged heat exposure but, even if it is not, as in the case of *M. bore* larvae, the delicate cost/benefit balance of staying active under extreme heat may be modified by various possible changes in the behavior of ant prey in response to the same thermal conditions (Cerdá and Retana 2000). This issue is worth further study.

In summary, using trap-building insects, we showed that their behavioral response to heat, that is, activity levels, may be connected to fitness-relevant traits, such as the body state and developmental time. We further demonstrate that some of these insects, such as antlions inhabiting sunlit areas, display extraordinary thermal resilience and may well be one of the terrestrial living organisms best adapted to withstand prolonged high heat. We encourage further studies of the thermal biology of antlions, as well as other trap-building insects, such as wormlions or caddisflies.

## Notes

Data availability: Analyses reported in this article can be reproduced using the data provided by Miler et al. (2020).
